# Epidemiology and Evolutionary Dynamics of High Pathogenicity Avian Influenza (HPAI) H5N1 in Bangladesh

**DOI:** 10.1155/2023/8499018

**Published:** 2023-07-12

**Authors:** Ariful Islam, Tasnim Ara, Emama Amin, Shariful Islam, Md. Abu Sayeed, Tahmina Shirin, Mohammad Mahmudul Hassan, Marcel Klaassen, Jonathan H. Epstein

**Affiliations:** ^1^EcoHealth Alliance, New York, NY 10018, USA; ^2^Centre for Integrative Ecology, School of Life and Environmental Sciences, Deakin University, Geelong, Victoria, VIC 3216, Australia; ^3^Institute of Epidemiology, Disease Control and Research (IEDCR), Dhaka 1212, Bangladesh; ^4^Queensland Alliance for One Health Sciences, School of Veterinary Science, The University of Queensland, Brisbane, QLD 4343, Australia; ^5^Faculty of Veterinary Medicine, Chattogram Veterinary and Animal Sciences University, Chattogram 4225, Bangladesh

## Abstract

Understanding disease clustering and transmission patterns improves the prevention and control of disease. Herein, we described the epizootic characteristics and spatiotemporal dynamics of High Pathogenicity Avian Influenza (HPAI) H5N1 outbreaks as well as clade diversity and phylodynamics of H5N1 over time and across host species in Bangladesh. We used Moran's I, Geary's C, Getis-Ord Gi^∗^, and a space-time permutation model to analyze the spatiotemporal patterns of H5N1 outbreaks. We used Bayesian phylogenetic analysis to generate a time-scaled maximum clade credibility (MCC) tree. Our study revealed nine HPAI H5N1 epizootic waves between 2007 and 2020 that invariably peaked in the wintertime. After vaccination of poultry against H5N1 was introduced in 2012, the incidence of HPAI H5N1 outbreaks and poultry mortality decreased significantly over time. Nonetheless, our research revealed that the virus continued circulating unabatedly in Bangladesh. The various spatiotemporal analyses were identified up to nine space-time clusters across Bangladesh, with the most significant clustering and hotspots of H5N1 outbreaks in and around the district of Dhaka. Since 2007, four H5N1 clades have been detected in Bangladesh, with only clade 2.3.2.1a continuing to circulate since 2011, which was followed up by the reassorted clade 2.3.2.1a in 2012. The HA gene of the H5N1 clade 2.3.2.1a has been reassorted into at least nine subgroups (R1–R9). After 2016, the first eight groups disappeared, with only the R9 group remaining. Spatiotemporal patterns and phylodynamics of H5N1 outbreaks are crucial for developing targeted and appropriate HPAI control and prevention measures. We recommended intensive monitoring of biosecurity measures and disease records in high-priority areas, along with assessing vaccine efficacy to better control HPAI outbreaks in Bangladesh.

## 1. Introduction

Since the virus was first discovered in 1996 in southern China, the global spread of High Pathogenicity Avian Influenza (HPAI) H5N1 in poultry and wild birds has posed a significant panzootic risk and a concern to public health [1]. Its spread outside Asia and the emergence of the HPAI H5N1 virus in Europe in the second half of 2005 underlined the magnitude of the pandemic threat [2]. In 2020, a new clade 2.3.4.4b of H5N1 emerged [3], increasingly spreading through the migratory birds and causing outbreaks in many parts of Africa, Asia, and Europe [4, 5]. By April 2023, the virus was discovered in a wide spatial area that includes more than 100 countries and islands across the world, with 8344 outbreaks in domestic and 5573 outbreaks in wild birds [6, 7]. In addition, there has been a spread to nonavian species [3]. Since its emergence, the virus has resulted in at least 873 laboratory-confirmed human cases in 21 countries [8].

HPAI H5N1 virus emerged in Bangladesh in 2007. Since then, 585 outbreaks have been recorded in 54 of Bangladesh's 64 districts, making it one of the countries with the highest number of reported cases worldwide [6, 9], challenging both the country's commercial and backyard poultry production systems [10–12]. Fortunately, there have been just eight human cases of H5N1 in Bangladesh to date. After the implementation of vaccination of poultry against HPAI H5N1 in Bangladesh in 2012, the virus continued to cause sporadic outbreaks in poultry in various locations of the country [13, 14]. In 2008 alone, due to HPAI H5N1 outbreaks in Bangladesh, more than 1.8 million chickens were culled, resulting in an estimated economic loss of US$ 40 million [9, 15].

Consequently, the control of HPAI in poultry is of paramount importance for Bangladesh. To this end, it is crucial to understand the dynamics of H5N1 outbreaks for future planning, decision-making, and acting. Studying spatiotemporal dynamics can, for instance, identify high-risk regions where monitoring and early detection efforts should be concentrated [16] and support the development of mitigation strategies such as culling or vaccination campaigns [17, 18]. The information is not only of relevance to Bangladesh, but also in building an understanding of spatiotemporal dynamics globally and how the virus is spreading across national boundaries and continents [19, 20]. Investigations into the spatiotemporal dynamics of HPAI H5N1 outbreaks may also include identifying disease clusters, which can also lead to better disease control and prevention efforts [21]. The space-time pattern of HPAI H5N1 outbreak clustering in Bangladesh could designate places where more research should be conducted to learn about the virus's entrenchment and propagation [22–24]. In zoonotic disease outbreaks worldwide, including HPAI H5N1, the significance of using space-time clusters has been demonstrated previously [23, 25–27]. Throughout Asia, significant space-time effects on the spread and severity of HPAI H5N1 outbreaks were found in China [28], South Korea [29], Vietnam [30–32], and Thailand [33]. Therefore, we conducted an analysis of the spatiotemporal dynamics of HPAI H5N1 outbreaks, including a range of geospatial tools to identify clusters and hotspots of HPAI H5N1 in Bangladesh. While several studies have previously reported on the epidemiology of HPAI H5N1 in Bangladesh, these were limited in the extent to which spatiotemporal dynamics were investigated and were also limited to the period prior to the onset of poultry vaccination against HPAI H5N1 in 2012 [10, 11], which was a paramount initiative to reduce the burden of HPAI H5N1 on Bangladesh's poultry industry.

Also, our understanding of the evolutionary dynamics of HPAI H5N1 in Bangladesh is still limited. What we know is that it was clade 2.2.2 HPAI H5N1 virus that was introduced into Bangladesh in February 2007, followed by clade 2.3.4.2 and 2.3.2.1a viruses in 2011 [34, 35]. Since the introduction of poultry vaccination in 2012, clades 2.2.2 and 2.3.4.2 of the HPAI H5N1 virus have disappeared; however, clade 2.3.2.1a has continued to evolve and cause outbreaks in Bangladesh despite the use of vaccination in commercial chicken farms [36–38]. Here, we combined these previous works and present a holistic overview of the phylodynamic of HPAI H5N1 sequences collected from the domestic chickens, waterfowl, and other wild birds in Bangladesh between 2007 and 2020 and explore the genetic diversity of HPAI H5N1 over time and across species in Bangladesh.

## 2. Methods and Materials

### 2.1. Data Sources and Processing

#### 2.1.1. HPAI H5N1 Outbreak Data

The livestock sector in Bangladesh is dominated by poultry farming and is increasing. In 2019, the annual per capita consumption of poultry meat in Bangladesh was 8.5 kg of meat and 5.1 kg of eggs [39]. Most of the HPAI outbreaks reported in Bangladesh have occurred in poultry farms [10]. HPAI outbreak reporting in Bangladesh relies on passive and active reporting. We tabulated all 585 reported HPAI outbreaks in Bangladesh from the Global Animal Disease Information System (EMPRES-i) database between 2007 and 2020, available at the Food and Agriculture Organization (FAO), which also includes data from the World Organization for Animal Health (WOAH) websites, between 2007-2020 [6, 9]. The EMPRES-i data was accessed on January 31, 2022. No outbreak data have been officially reported to FAO and WOAH by Bangladeshi authorities after 2018. FAO reports outbreak data, including subtype, pathogenetic status, detection date, and geographical location, from which we determined the district (*n* = 64) in Bangladesh in which the outbreak took place using digital maps from the open-source DIVA-GIS website [40].

#### 2.1.2. H5N1 Sequence Data

On May 23, 2022, we retrieved all accessible HA gene sequences of H5N1 with respective epidemiological metadata (*n* = 413) from the GISAID Epiflu database [41]. We also accessed the national center for biotechnology information (NCBI) influenza virus database [42] for cross-checking but found no additional HA gene sequences for Bangladesh. For all sequences, we collected corresponding metadata from GISAID supplemented with other published information from published articles and other publicly available sources. These data included geospatial data and information on whether the host was from the wild or domestic and the type of production system (backyard or commercial) from which it came. These data were used to supplement the outbreak data from EMPRES-i. For the building of phylogenetic trees, all sequences from 2007 to 2020 were downloaded.

### 2.2. Data Analysis

#### 2.2.1. Description of the Temporal and Spatial Distribution of HPAI H5N1 Incidences

The monthly number of HPAI H5N1 outbreaks across Bangladesh, as well as the number of affected districts across the nation and incidences based on metadata associated with published sequences, was presented as a time plot to show the temporal dynamics of the HPAI H5N1 outbreaks. The distribution of outbreaks across species and production systems (i.e., backyard or commercial farm) was demonstrated using prevalence, including a 95% binomial exact confidence interval of the prevalence [43]. During epizootic waves, the frequency of disease outbreaks rises quickly and then progressively falls towards the end of the epizootic [17]. To detect HPAI H5N1 outbreak epizootic waves, we considered the endpoint for the epizootic curve to be reached if there were no reported outbreaks in two consecutive months [44].

For spatial presentations and data analyses, we considered four time periods, i.e., all outbreaks from 2007–2018, as well as the 4-year periods 2007–2010, 2011–2014, and 2015–2018. We visualized the point distribution of HPAI H5N1 outbreaks in a digitized shape file of Bangladesh using ArcMap 10.8.

Adaptive kernel density estimation is a method for estimating the first-order features of a spatial stochastic process reflecting a global or large-scale trend [45], which we used to create a smoothed presentation of how the distribution and frequency of outbreaks varied across the nation. The kernel density is estimated as follows. Let (*x*_1_, *x*_2_,…, *x*_*n*_) be independent and identically distributed samples drawn from some univariate distribution with an unknown density *f* at any given point *x*. We are interested in estimating the shape of this function *f*. Its kernel density estimator is as follows:

(1) $$\begin{array}{c} {\hat{f}}_{h}\left(x\right)=\frac{1}{n}\overset{n}{\underset{i=1}{\sum }}K_{h}\left(x-x_{i}\right)=\frac{1}{nh}\overset{n}{\underset{i=1}{\sum }}K\left(\frac{x-x_{i}}{h}\right)\text{,} \end{array}$$

where *K* is the kernel, a non-negative function, and *h* > 0 is a smoothing parameter called the bandwidth.

#### 2.2.2. Statistical Analyses of Global Spatial Patterns in HPAI H5N1 Outbreaks

We used Global Moran's I index [24], Global Geary's C [46], and Global Getis-Ord Gi^∗^ [24] to analyze the spatial patterns in HPAI H5N1 outbreaks. A Moran's I value close to zero indicates a random distribution, while a negative Moran's I value indicates more dispersed H5N1 outbreak sites from random and positive values which indicate a clustering of H5N1 outbreaks. Moran's I value is calculated using the following equation:

(2) $$\begin{array}{c} I=\frac{n}{s_{0}}\frac{\sum_{i=1}^{n}\sum_{j=1}^{n}w_{{i,j}^{,}}x_{i}x_{j}}{\sum_{i=1}^{n}x_{i}^{2}}\text{,} \end{array}$$

where *x*_*i*_ and *x*_*j*_ are the deviations of an attribute for feature *i* and *j* from their respective means, *w*_*i*,*j*_ is the spatial weight between features *i* and *j*, *n* is equal to the total number of features, and *S*_o_ is the aggregation of all spatial weights.

Geary's *C* measures spatial autocorrelation with values between 0 and 1 indicating positive and values higher than 1 indicating negative autocorrelation is calculated as follows:

(3) $$\begin{array}{c} C=\frac{\left(N-1\right)\sum_{i}\sum_{j}w_{ij}{\left(x_{i}-x_{j}\right)}^{2}}{2W\sum_{i}{\left(x_{i}-\bar{x}\right)}^{2}}\text{,} \end{array}$$

where *N* is the number of spatial units indexed by *i* and *j*, *x* is the variable of interest, $\bar{x}$ is the mean of *x*, *w*_*ij*_ is a matrix of spatial weights with zeroes on the diagonal (i.e., *w*_*ii*_ = 0), and *W* is the sum of all *w*_*ij*_.

Finally, Global Getis-Ord Gi^∗^ values that are negative identify cold spots and values that are positive identify hot spots or areas where there are particularly many outbreaks. Using the parameters used in the calculation presented above, this statistic is calculated using the following equation:

(4) $$\begin{array}{c} G=\frac{\sum_{i=1}^{n}\sum_{j=1}^{n}w_{ij}x_{i}x_{j}}{\sum_{i=1}^{n}\sum_{j=1}^{n}x_{i}x_{j}}\text{.} \end{array}$$

#### 2.2.3. Statistical Analyses of Local Spatial Patterns in HPAI H5N1 Outbreaks

Aside from global spatial statistics, local spatial statistics were also calculated to identify specific areas with particularly high or low numbers of outbreaks. The local Moran's I statistic is calculated as follows:

(5) $$\begin{array}{c} I_{i}=\frac{x_{i}-\bar{x}}{S_{i}^{2}}\overset{n}{\underset{i=1,j\neq 1}{\sum }}\omega_{i,j}\left(x_{j}-\bar{x}\right)\text{,} \end{array}$$

where *x*_*i*_ is the attribute feature of *i*, $\bar{x}$ is the mean of the corresponding attribute, *ω*_*i*,*j*_ is the spatial weight between features *i* and *j*, with *n* being the total number of functions, and

(6) $$\begin{array}{c} S^{2}=\frac{\sum_{j=1,j\neq i}^{N}{\left(x_{j}-\bar{x}\right)}^{2}}{n-1}\text{.} \end{array}$$

The value of local Moran's I range from +1, indicating high-high (HH) or low-low (LL) clusters, through 0 (= *a* random pattern) to −1, indicating high-low (HL) or low-high (LH) outliers. Thus, a HH (LL) cluster denotes several neighboring areas with a relatively high (low) incidence of outbreaks, while a HL (LH) outlier is a high (low) value surrounded by a region with a predominantly low (high) outbreak frequency.

The local Getis-Ord Gi^∗^ statistic is calculated using the following equation:

(7) $$\begin{array}{c} G_{i}^{*}=\frac{\sum_{j=1}^{n}w_{i,j}\;x_{j}-\bar{X}\sum_{j=1}^{n}w_{i,j}}{S\sqrt{\left(\left[n\sum_{j=1}^{n}w_{i,j}^{2}-{\left(\sum_{j=1}^{n}w_{i,j}\right)}^{2}\right]/n-1\right)}}\text{,} \end{array}$$

where *x*_*j*_ is the attribute value for feature *j*, *w*_*i*,*j*_ is the spatial weight between feature *i* and *j*, *n* is equal to the total number of features and the interpretation of the values is similar to that of the Global Getis-Ord Gi^∗^ statistic.

#### 2.2.4. Space-Time Clustering Detection Using Space-Time Permutation Model

Using the SaTScan 8.2.1 software, retrospective space-time permutation scan statistics were used to determine the spatiotemporal clusters for HPAI H5N1 outbreaks [23] for the periods 2007–2010, 2011–2014, and 2015–2018. In this analysis, we largely followed the procedure as outlined in [27] using a temporal scanning window of a maximum of 2 years within each four-year period and a spatial scanning window that allowed a maximum of 50% of the outbreaks.

#### 2.2.5. Bayesian Phylogenetic Analysis

We used MEGA 11 to align the sequences (https://www.megasoftware.net) and identify sequences with artifacts that were eliminated. Ultimately, 274 sequences were retained for analyses. We constructed the Bayesian phylogenetic tree across all sequences using BEAST v.1.10.4 (https://beast.community) with an uncorrelated lognormal relaxed molecular clock. The gamma-distributed rate variation among sites with four rate categories (HKYþG) [47] was used. The Markov Chain Monte Carlo (MCMC) was run for 50 million steps, and the trees and the parameters were sampled every 5,000 steps. Tree Annotator (https://beast.community/treeannotator) was used to generate time-scaled maximum clade credibility (MCC) tree in BEAST and was visualized using Figtree 1.4.3 (http://tree.bio.ed.ac.uk/software/figtree/). We also constructed a phylogenetic tree for 289 sequences from 2011-2020 belonging to clade 2.3.2.1a following the same methodology as described above for all sequences combined.

## 3. Results

### 3.1. Description of Temporal and Spatial Distribution of HPAI H5N1 Incidences

**Figure 1:**
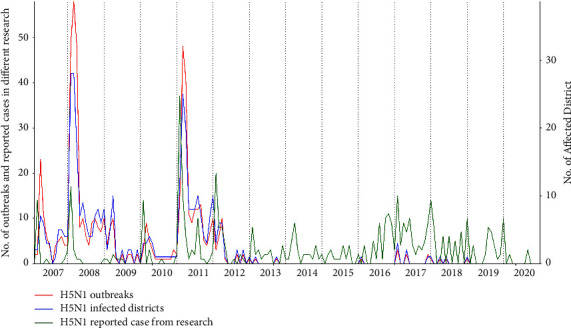
Temporal trends in the number of monthly HPAI H5N1 outbreaks (red line), number of districts where outbreaks took place (blue line), and reported cases based on published sequence data (green line) from 2007 to 2020.

There have been 585 H5N1 outbreaks in poultry and wild birds since the first incidence was identified in 2007. The vast majority, i.e., 98.8% (95% CI: 97.6–99.5) of the outbreaks, concerned chickens, with only 1.2% (95% CI: 0.5–2.5) of the outbreaks in house crows (*Corvus splendens*). Furthermore, most outbreaks in chickens were reported among commercial chickens (89%), followed by backyard chickens (9.7%) (Table 1). The temporal dynamics of HPAI H5N1 outbreaks in Bangladesh (Figure 1), show two major peaks in February 2008 and January 2011, with 58 and 49 outbreaks, respectively. After vaccination against HPAI H5N1 was introduced at the start of 2012, the number of cases dropped dramatically. Although the official reporting of outbreaks stopped in 2018, H5N1 cases were still detected in 2019 and 2020 based on metadata related to published sequences (green line in Figure 1).

**Figure 2:**
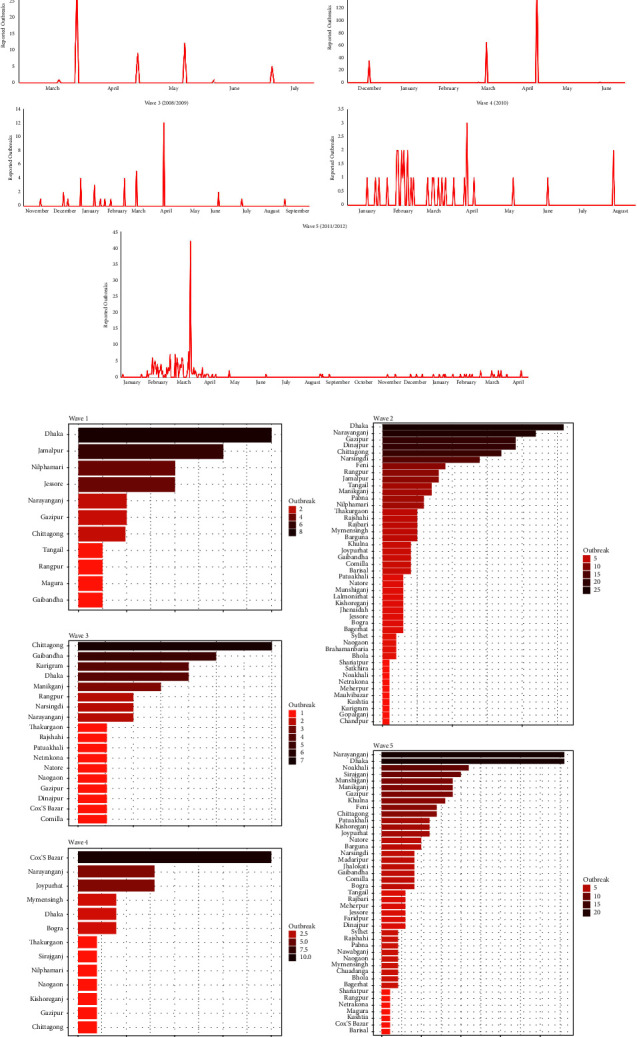
The first five and major HPAI H5N1 outbreak waves in Bangladesh. (a) Temporal distribution of outbreaks during each wave and (b) distribution of outbreaks across districts in each wave.

Nine waves of HPAI H5N1 infections can be decerned between 2007 and 2020.  Table 2 enumerates all waves, along with the number of documented outbreaks. All waves were centered around wintertime in Bangladesh. The first 5 waves were the largest, concerned chickens exclusively, and occurred annually between the first occurrence of HPAI H5N1 in Bangladesh and the moment vaccination against HPAI H5N1 was introduced.  Figure 2(a) demonstrates the temporal distribution of these 5 epizootic waves in more detail, while Figure 2(b) depicts the spatial scale as the frequency of outbreaks across the impacted districts. The latter shows Dhaka to be the most affected district by wave 1 and 2, followed by Chittagong, Cox's Bazar, and Narayanganj in waves 3, 4, and 5. The remaining four small waves, while not occurring annually, remained centered around wintertime and foremostly concerned outbreaks amongst house crows.

**Figure 3:**
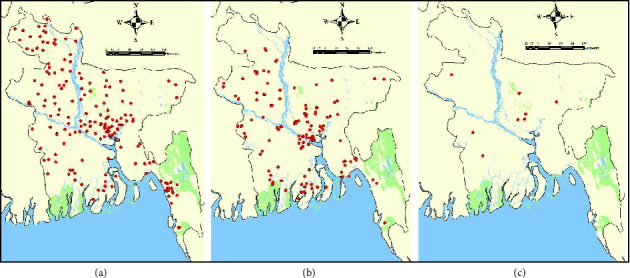
Spatial distribution of HPAI H5N1 outbreaks in Bangladesh over the periods (a) 2007–2010, (b) 2011–2014, and (c) 2015–2018.

The spatial distribution of the 585 HPAI H5N1 outbreaks in Bangladesh and how it changes over time are presented in more detail in Figure 3. The maps depict that H5N1 outbreaks concentrated in and around Dhaka district, also when the number of outbreaks decreased over time throughout the country.

**Figure 4:**
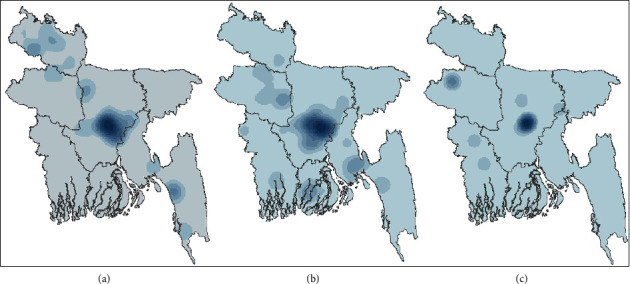
Adaptive kernel density estimation (KDE) of HPAI-H5N1 outbreaks in Bangladesh over the periods (a) 2007–2010, (b) 2011–2014, and (c) 2015–2018.

The adaptive kernel density estimation for the HPAI H5N1 outbreaks in each of the three-time periods (Figure 4) changes little in locations over time, with a major concentration around the Dhaka district. This confirms the impression already provided by Figure 3, including the decrease in intensity of the kernel density in the 2015 to 2018 period. Overall, outbreaks occurred along an oblique line that ran from southeast to northwest through the country.

### 3.2. Statistical Analyses of Spatial Patterns in HPAI H5N1 Outbreaks

Confirming the impression of this nonrandom distribution and clustering of outbreaks across the country, global Moran's I was significant and positive across the entire period 2007–2018 as well as the four-year interval (Table 3). In line with this, global Geary's C values tended to be lower than 1 across most periods, suggesting spatial autocorrelation in the outbreak, but in none of the cases this was significant (Table 3). Finally, again in line with the abovementioned, global Getis-Ord Gi^**∗**^ values are significantly positive, suggesting the existence of outbreak hot spots for all investigated periods (Table 3).

**Figure 5:**
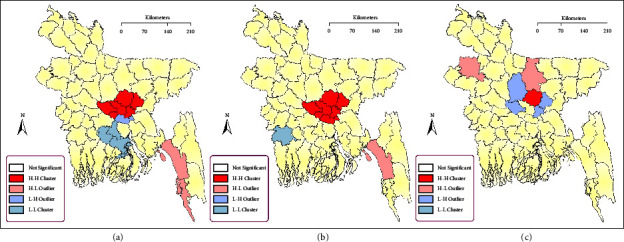
Spatial cluster detection of HPAI H5N1 outbreaks using local Moran's I for the periods (a) 2007–2010, (b) 2011–2014, and (c) 2015–2018.

The abovementioned significant Global Moran's I and Getis-Ord Gi^∗^ spatial statistics call for a more in-depth, local analysis identifying the districts that stand out and ultimately cause the global, nonrandom distribution of outbreaks. In doing so, we found that from 2007 to 2010, the local Moran's I had greater values in Dhaka, Gazipur, Manikganj, Narayanganj, and Narsingdi. From 2011 to 2014, a similar pattern was observed, with a greater value in Dhaka, while Madaripur was added to the cluster. Finally, from 2014 to 2018, in Gazipur, the significance of the H5N1 cluster decreased and the local Moran's I values decreased dramatically (Figure 5).

**Figure 6:**
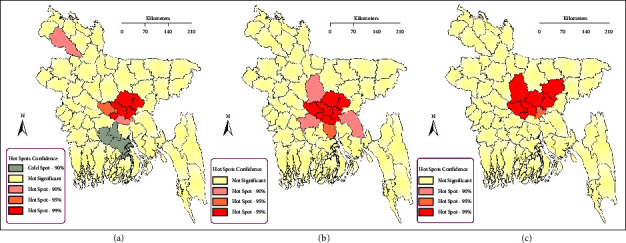
Hotspot detection of HPAI H5N1 outbreaks using Getis-ord general G^∗^ for the period (a) 2007–2010, (b) 2011–2014, and (c) 2015–2018.

Regarding Getis-Ord General Gi^∗^, the “local” variant of global Getis-Ord Gi^∗^, between 2007 and 2010, Dhaka, Narayanganj, Gazipur, Narayanganj, and Narsingdi were identified as H5N1 epizootic hotspots with 99% confidence, followed by Manikganj (95% confidence) and Munshiganj and Dinajpur (90% confidence). For the period 2011 and 2014, these were Dhaka, Gazipur, Manikganj, Munshiganj, Narayanganj, and Narsingdi (99% confidence), Shariatpur (95% confidence), and Comilla, Tangail, and Faridpur (90% confidence). Finally, between 2015 and 2018, Dhaka, Gazipur, Kishoreganj, Manikganj, Narsingdi, and Tangail (99% confidence) and Narayanganj (95% confidence) were identified as H5N1 hotspots (Figure 6).

**Figure 7:**
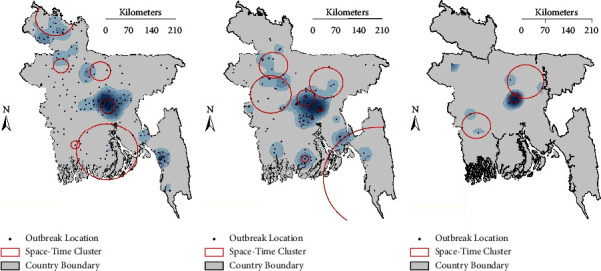
Space-time clusters of HPAI H5N1 outbreaks detected using space-time permutation scan statistics for the periods (a) 2007–2010, (b) 2011–2014, and (c) 2015–2018. Black dots represent outbreaks, red circles represent the identified clusters, and blue shading represents the outbreak density from adaptive Kernel density estimation (cf. Figure 4).

Following on from the significant Moran's I and Getis-Ord G^∗^ results, we used space-time permutation scan statistics to identify clusters of HPAI H5N1 outbreaks across the country for the three 4-year periods (Table 4; Figure 7). The most crucial cluster (within ≤1 km radius) was found in Ramu, Cox's bazar, followed by Araihazar, Narayanganj, and Sarishabari, Jamalpur. The largest cluster, with a radius of 154.7 kilometers, was formed between June 1 and September 30, 2011, with the centroid in Ramu, Cox's Bazar.

### 3.3. Phylodynamic and Clade Diversity of H5N1 Virus in Bangladesh

**Figure 8:**
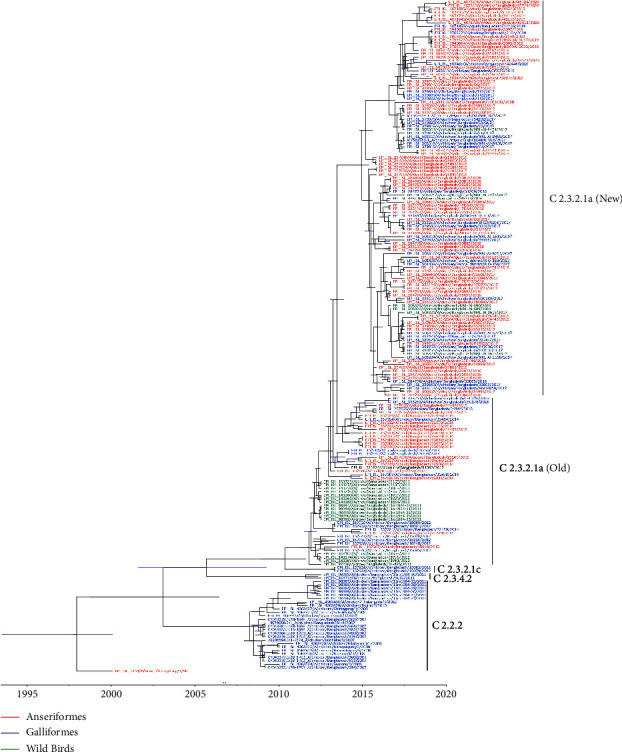
The maximum clade credibility (MCC) temporal phylogeny of the hemagglutinin (HA) gene of the H5N1 virus by host type in Bangladesh from 2007–2020. Different colors denote H5N1 viruses from different hosts, and the thickness of branches represents the posterior probabilities of the ancestral host type. The red color indicates domestic anseriformes hosts, blue indicates domestic Galliformes hosts, and green indicates wild bird host.

**Figure 9:**
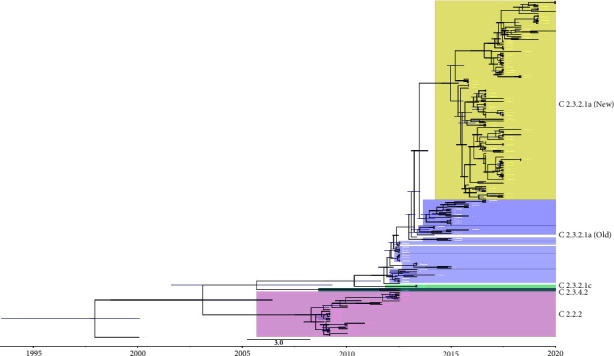
The compressed Bayesian phylogenetic MCC tree of the HA gene of the H5N1 virus in Bangladesh from 2007 to 2020 (cf.  Figure 8), highlighting the temporal pattern of the circulating clades. Each clade is denoted using a different color.

Following a comprehensive phylogenetic analysis of HA gene sequences of H5N1 HPAI virus isolates from Bangladesh, four different clades of H5N1 viruses were discovered (Figures 8 and 9). Clade 2.2.2 was introduced in Bangladesh in 2007 and resulted in the formation of two major lineages, one in 2007-2008 and the other in 2007-2011. In 2011, clade 2.3.4.2 and in 2012, clade 2.3.2.1c were introduced in Bangladesh and formed a monophyletic cluster. Clade 2.3.2.1a has been detected in Bangladesh since 2011. Since 2012, no new cases of HPAI H5N1 viruses of clades 2.2.2, 2.3.4.2, and 2.3.2.1c have been detected in Bangladesh. A reassortment occurred inside the subclade 2.3.2.1a in 2015, resulting in a new group, which we will refer to as clade 2.3.2.1a (new) emerging from clade 2.3.2.1a (old) from hereon. Galliformes were prominent hosts in clades 2.2.2, 2.3.4.2, and 2.3.2.1c, but clade 2.3.2.1a exhibited significant variation in host species. The marked host diversity was seen in both groups (i.e., both new and old) within this clade 2.3.2.1a.

**Figure 10:**
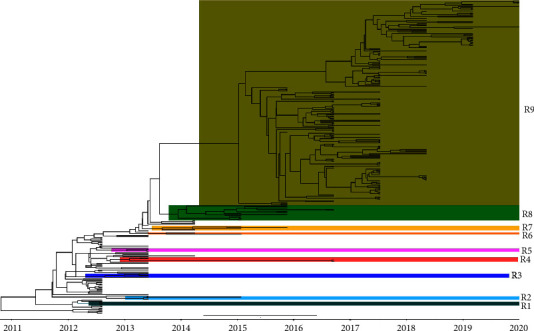
Bayesian phylogenetic MCC tree of HA gene of clade 2.3.2.1a H5N1 virus in Bangladesh. The clade has been divided into genetic subgroups R1–R9 based on posterior probability scores greater than 99% using different colors.

The time-scaled phylogenetic tree of HA genes indicated that H5N1 clade 2.3.2.1a viruses in Bangladeshi avian hosts (domestic Anseriformes, domestic Galliformes, and wild birds) diverged into at least nine genetic subgroups (R1–R9), as supported by a high posterior probability (>99%) in the MCC tree (Figure 10). After 2016, most subgroups (R1–R8) were not observed and replaced by viruses belonging to the R9 subgroup.

## 4. Discussion

We investigated the spatiotemporal patterns and evolutionary dynamics of HPAI H5N1 outbreaks in poultry and wild birds in Bangladesh between 2007 and 2020. Most outbreaks occurred in the January-March period when Bangladesh's ambient temperatures are typically at their lowest. This broadly corresponds with the period also designated as high-risk in other Southeast Asian countries [30, 48, 49] and might relate to higher survival of avian influenza viruses in colder environments when outside a host [50, 51]. However, our phylodynamic analyses clearly indicate that HPAI H5N1 viruses continued circulating during the summer periods and can be considered endemic in Bangladesh.

The nine identified waves differed in length, the timing of peak incidence and number of outbreaks involved. Aside from these wave variations being possibly due to variations in surveillance intensity and sensitivity, they also may have been due to differences in response to each outbreak wave and differences in disease dynamics [15]. Regarding the latter, wave 5 in 2011 coincided with the introduction of a novel H5 clade 2.3.2.1 in Bangladesh. The advent of this virus may have changed transmission dynamics, such as the severity of infection as a percentage of flock size, in contrast to viruses prevalent in 2008 [44]. While vaccination of chickens in commercial poultry farms, which commenced in early 2012 towards the end of the fifth wave, resulted in a dramatic decrease in HPAI H5N1 outbreaks across Bangladesh, based on published sequence data, we concluded that HPAI H5N1 continued to be present all over the country. This is yet another reason to consider HPAI H5N1 to be endemic in Bangladesh. Although most outbreaks occurred in poultry and mainly in chicken, our study revealed that approximately 1% of outbreaks occurred in wild birds, especially house crows. That wild birds may get infected with avian influenza but subsequently recover has previously been concluded based on the occurrence of antibodies against avian influenza in blood samples of wild birds in Bangladesh [52]. Waves 7–9 were dominated by house crow mortalities, and these could be connected to live bird markets (LBMs). Several subtypes of avian influenza virus, including HPAI viruses, are circulating in LBMs, and scavenging house crows may get infected with H5N1 during feeding on offal at LBMs [53–55].

### 4.1. Geospatial Modeling of H5N1 Outbreaks in Bangladesh

We used exploratory and analytical geospatial statistics to characterize the spatiotemporal pattern in HPAI H5N1 outbreaks in chicken and wild birds in Bangladesh from 2007 to 2018. These analyses showed that outbreaks were centered in and around the Dhaka district, supporting observations from the previous studies [10, 11]. Dhaka has the highest human population density in the country. Human activity can substantially influence the spread of infectious diseases [15, 56], with human population density typically being associated with high poultry production and trading, increasing cross-infection risk [57]. There is strong demand for chicken products in the metropolitan city of Dhaka, where poultry populations from different parts of the country are congregated in LBMs [58], which are known to act as hubs for AIV transmission [53]. Road networks have previously been considered to be important in explaining the spread of HPAI H5N1 [33, 59, 60]. Using space-time permutation modeling, we found clusters in the Dhaka, Bogura, and Barisal region from 2007 to 2010, with large cities (Ramu, Cox's Bazar, and Araihazar, Narayanganj) apparently increasing the risk of viral propagation in the surrounding areas [27]. The intensity of H5N1 outbreak occurrence was highest in 2007–2010, followed by 2011–2014, and then the lowest intensity was observed in 2015–2018, due to vaccination programs against the H5N1 virus in commercial poultry starting in 2012 [61].

### 4.2. Evolution and Transmission Dynamics of H5N1 Subtypes and Their Clade Diversity in Bangladesh

Our phylodynamic analysis shows that the H5N1 viruses isolated from poultry and wild birds in Bangladesh between 2007 and 2020 cover four clades, namely 2.2.2, 2.3.4.2, 2.3.2.1c, and 2.3.2.1a (Figure 9), which is in accordance with the findings of previous studies conducted in Bangladesh [62–64]. Clade 2.2.2 was first detected in poultry in the region (i.e., India and Pakistan) in 2006 [65]. It was introduced in Bangladesh in 2007 and triggered early epizootics in Bangladesh. This clade 2.2 virus, which may have originated from a single introduction, circulated in Bangladesh between 2007 and 2010 and was responsible for a large number of outbreaks with severe impact on the poultry industry in Bangladesh [66–68]. Aside from causing havoc in the poultry industry, three human cases caused by clade 2.2.2 viruses were also recorded [62, 66]. In 2011, clades 2.3.4.2 and 2.3.2.1c were introduced in Bangladesh for a short period and created a monophyletic cluster [62, 67]. It has been suggested that clade 2.3.2.1c H5N1 has likely descended from a virus isolated from a Muscovy duck in Vietnam [69, 70], while clade 2.3.4.2 H5N1 is most likely a descendant of 2009, environment-isolated virus from Guizhou, China [61]. After the vaccination program started in 2012, clades 2.2.2, 2.3.2.1c, and 2.3.4.2 disappeared and have not been detected in Bangladesh since 2012 [37]. It is worth noting, though, that while there has not been an epizootic since 2012, the virus is still present. Clade 2.3.2.1a has regularly been detected since 2011. This clade has evolved into several subgroups, and according to our analysis, this clade comprises at least nine subgroups (R1-R9). Of these, only subgroup R9 has been detected since 2016. Barman et al. [63] and Barman et al. [71] also reported that clade 2.3.2.1a is dividing into subgroups and evolving rapidly, which is alarming because reassortment and genetic drift may result in the emergence of a new clade and widen the host reservoir. Clade 2.3.2.1a has also been detected in Bhutan and Vietnam [72, 73]. While Galliformes were prominent in clades 2.2.2, 2.3.2.1c, and 2.3.4.2, domestic Anseriformes, particularly domestic ducks, acted as clade 2.3.2.1a's major host species [35] from which the virus next spread to domestic Galliformes in 2013. Domestic ducks are frequently raised in Bangladesh in the backyard or on small-scale commercial farms. Domestic ducks in Bangladesh are highly susceptible to H5N1 infection, shedding large amounts of the virus, which may contaminate the environment and possibly infect other poultry or wild birds. The H5N1 vaccination program in Bangladesh was concentrated on the commercial layer and breeder hens, with domestic ducks being a secondary target for vaccination only [61]. Possibly as a result, Anseriformes have initially emerged as the main host species for clade 2.3.2.1a in Bangladesh, with its wide spread of clade 2.3.2.1a in domestic duck flocks being facilitated by continuing mutations [74].

The new reassorted 2.3.2.1a H5N1 viruses present in group R9 contain the hemagglutinin, neuraminidase, and matrix genes of the ancestral R8 group and other internal genes of LPAI Eurasian-lineage of AIV that are closely related to LPAI viruses isolated from nomadic domestic ducks and wild birds in a wetland region of northeastern Bangladesh. In this area, domestic ducks have frequent contact with migratory waterfowl, and this interface may have played a crucial role in the emergence of this novel genotype of highly pathogenic H5N1 viruses [63, 71, 75]. Since 2011, in Bangladesh, clade 2.3.2.1a has been detected and reassorted in multiple host species, including chickens, ducks, geese, migratory wild waterfowl, quail, pigeons, and house crows [63, 74, 76]. The fact that the current HPAI H5N1 clade 2.3.2.1a (new) is continuously evolving and widening its host reservoir, including spillover into mammalian hosts, including humans, in Bangladesh, is a worrying development. It underscored the need for continuing genomic surveillance and improved biosecurity measures to prevent new viral introductions and the spread between farms and other entities of the Bangladeshi poultry industry.

For our overview presented here, we are confident that our data collation includes the most outbreak data. We cannot rule out some underreporting of outbreaks due to fear of compulsory culling and insufficient financial compensation. Despite these possibilities, it is unlikely that any missing data would lead to biases in the patterns observed. Our study therewith provides an in-depth description of the spatiotemporal and evolutionary dynamics of H5N1 outbreaks in Bangladesh, which we hope will serve as a foundation for further studies aimed at improving our understanding of the drivers of HPAI H5N1 outbreaks, which can be used in the development of effective disease control strategies in Bangladesh.

## 5. Conclusion

Our research explored the spatiotemporal dynamics of HPAI H5N1 outbreaks and the genetic evolution and clade diversity of the virus in Bangladesh. Our study identified nine HPAI H5N1 epizootic waves between 2007 and 2020. We observed a significant decrease in the number of outbreak reports after the onset of HPAI H5N1 vaccination for commercial poultry in 2012. Nevertheless, post-2012 surveillance data reveals year-round presence of the H5N1 virus in farms and LBMs, indicating the virus is endemic in Bangladesh. The application of spatiotemporal analyses showed the presence of HPAI H5N1 outbreak hotspots, with densely populated areas being most vulnerable to outbreaks, and providing crucial information for the development of effective disease control and prevention strategies. Since 2012, and despite a national HPAI H5N1 vaccination campaign, clade 2.3.2.1a dominates the avian influenza landscape in Bangladesh, with strains from this clade being found in an increasing number of species, including mammals. In the face of this worrisome proliferation, we recommend continued monitoring of the evolutionary dynamics of HPAI H5N1, implementing stringent biosecurity measures in both LBMs and farms, and the monitoring of vaccine efficacy to put a stop to this rise of HPAI H5N1 and prevent incursions of novel strains of H5Nx viruses into Bangladesh.

## Figures and Tables

**Table 1:** H5N1 incidence across species and production systems.

Species	Frequency of outbreaks	%95% CI
All poultry	578	98.8 (97.6–99.5)
Backyard chicken	57	9.7 (7.5–12.4)
Commercial chicken	521	89.1 (86.2–91.5)
House crow	7	1.2 (0.5–2.4)

**Table 2:** Waves of HPAI H5N1 outbreaks in Bangladesh.

Wave	Start of wave	End of wave	Reported outbreaks
1	March 2007	July 2007	32
2	December 2007	June 2008	258
3	November 2008	September 2009	39
4	January 2010	August 2010	31
5	January 2011	April 2012	209
6	October 2012	March 2013	4
7	February 2016	February 2016	1
8	January 2017	May 2017	5
9	December 2017	June 2018	6

**Table 3:** Geospatial statistical analyses results of HPAI H5N1 outbreaks across four different periods between 2007 and 2018 across the whole of Bangladesh.

Year	Global Moran's I	Global Geary's C	Global Getis-ord Gi^∗^
	Statistic (P)	Statistic (P)	Statistic (P)
2007–2010	0.313 (<0.001)	0.863 (0.175)	0.142 (<0.001)
2011–2014	0.338 (<0.001)	0.840 (0.159)	0.148 (<0.001)
2015–2018	0.172 (0.002)	1.050 (0.590)	0.310 (<0.001)
2007–2018	0.365 (<0.001)	0.817 (0.135)	0.143 (<0.001)

**Table 4:** Space-time cluster detection using space-time permutation model of H5N1 outbreaks.

Year	Cluster number	Centroid location of cluster	Centroid latitude	Centroid longitude	Radius (km)	Date	O/E^∗^	*p* value
2007–2010	1	Ramu, Cox's bazar	21.481740	92.107820	<1	Feb 1, 2010, to Feb 28, 2010	31.1	<0.01
	2	Dimla, Nilphamari	26.175239	88.981384	52.72	May 1, 2007, to Aug 31, 2007	10.1	<0.01
	3	Gabtali, Bogura	24.891356	89.465322	21.02	Mar 1, 2010, to May 31, 2010	33.3	<0.01
	4	Araihazar, Narayanganj	23.780362	90.672508	<1	Nov 1, 2010, to Nov 30, 2010	120.0	<0.01
	5	Bakerganj, Barisal	22.552176	90.386798	85.6	Jan 1, 2008, to Jan 31, 2008	4.7	<0.01
	6	Badda, Dhaka	23.78300	90.45200	21.2	Feb 1, 2007, to Apr 30, 2007	3.5	<0.01
	7	Batiaghata, Khulna	22.719879	89.526707	11.9	Aug 1, 2008, to Aug 31, 2008	40.0	0.20
	8	Sarishabari, Jamalpur	24.719215	89.836450	<1	Jan 1, 2007, to Mar 31, 2007	8.9	0.30
	9	Mymensingh	24.75784	90.4892	28.3	Apr 1, 2008, to May 31, 2008	6.7	0.7

2011–2014	1	Mirjagonj, Patuakhali	22.340000	90.210000	9.9	Jan 1, 2012, to Mar 31, 2012	9.3	0.1
	2	Ullah Para, Sirajganj	24.2700	89.4300	56.2	Dec 1, 2011, to Dec 31, 2011	5.6	0.6
	3	Mahadebpur, Naogaon	24.90604	88.72794	39.0	Mar 1, 2012, to Dec 31, 2012	5.3	0.7
	4	Nikli, Kishoreganj	24.338586	90.924995	45.8	July 1, 2011, to Sep 30, 2011	5.2	0.7
	5	Feni Sadar	23.017880	91.432200	<1	Mar 1, 2012, to Aug 31, 2012	12.3	0.9
	6	Sonargaon, Narayanganj	23.699800	90.636400	6.3	Feb 1, 2013, to Sep 30, 2013	4.6	0.9
	7	Ramu, Cox's bazar	21.48174	92.10782	154.7	Jun 1, 2011, to Sep 30, 2011	4.5	0.9
	8	Kaliakair, Gazipur	24.010000	90.240000	24.9	Mar 1, 2011, to Apr 30, 2011	4.0	0.9

2015–2018	1	Serajdikhan, Munshiganj	23.5400	90.3900	14.1	Apr 1, 2017, to Nov 30, 2017	2.4	0.5
	2	Jashore	23.182367	89.190183	39.6	Mar 1, 2018, to Dec 31, 2018	4.0	0.8
	3	Kishoreganj	24.448812	90.662841	49.8	Dec 1, 2017, to May 31, 2018	4.0	0.8

Note: ∗O/E: observed/expected.

## Data Availability

The data used in the analyses can be obtained from the corresponding author upon reasonable request.
